# Photovoltaic water electrolysis reaching 31.3% solar-to-H_2_ conversion efficiency under outdoor operating conditions

**DOI:** 10.1038/s44172-026-00610-x

**Published:** 2026-04-27

**Authors:** Juan F. Martínez, Jens Ohlmann, Tom Smolinka, Frank Dimroth

**Affiliations:** Fraunhofer Institute for Solar Energy Systems ISE, Freiburg, Germany

**Keywords:** Solar cells, Hydrogen energy

## Abstract

Hydrogen generation from renewable energy sources allows balancing the intermittent nature of solar and wind power. The chemical energy stored in hydrogen can be efficiently converted back to electricity using fuel cells or hydrogen can be used in chemical processes or as secondary energy carrier in heat and gas markets. Several approaches have been investigated but most of them have a low conversion efficiency. Here we present a high-performance photovoltaic/electrolysis module that splits water molecules using the photovoltage of multi-junction solar cells. A Fresnel lens array concentrates direct sunlight onto photovoltaic cells with an open-circuit voltage above 4 V. These solar cells are electrically interconnected to the cathode and anode of two series-connected polymer electrolyte membrane electrolysis cells. A demonstrator with a lens area of 64 cm^2^ was measured outdoors, converting up to 31.3% of sunlight energy into chemical energy according to the higher heating value of hydrogen.

## Abstract (web summary)

Juan F. Martínez and colleagues present a hybrid concentrator photovoltaic powered polymer electrolyte membrane (PEM) electrolyzer operating under realistic outdoor conditions. The 64 cm^2^ module reached a world record solar-to-H_2_ conversion efficiency of 31.3% according to its higher heating value.

## Introduction

Climate change demands for substantial and fast defossilization of our energy supply systems. Solar and wind power provide renewable electricity at low cost, but their intermittent nature requires a highly flexible supplement to balance surplus generation and demand in times of insufficient generation^1^. Hydrogen as secondary energy carrier is discussed as a solution for matching demand and supply in different sectors due to its high specific energy of 39.4 kWh/kg being nearly three times that of natural gas (14.9 kWh/kg)^2,3^. When 1 kg of H_2_ reduces 7.9 kg of oxygen to form pure water, the reaction releases 39.4 kWh of heat according to its higher heating value (HHV). According to its lower heating value, a maximum of 33.3 kWh could be converted into electricity, e.g. using a fuel cell^4^. Translated into voltages at standard temperature and pressure, i.e., 0 °C and 1 bar, the latter corresponds to the reversible open cell voltage (V_rev_ = 1.23 V accounting for the change in Gibbs free energy of reaction) and the former to the thermo-neutral cell voltage (V_th_ = 1.48 V in addition accounting for the entropy losses)^5^. In the following, we use the thermo-neutral voltage in our efficiency calculations because this is the total amount of energy stored in the chemical bond of a H_2_ molecule regardless of its usage afterwards. Nevertheless, care should be taken when comparing with solar-to-H_2_ (STH) conversion efficiencies reported using the low heating value of H_2_ as these are 16.9% smaller.

Although electrolyzing green H_2_ with renewables is more expensive than reforming natural gas into gray H_2_ today, the expectation is that the former breaks even in regions with high solar resource by 2030^6^. In the long term, a study by the International Energy Agency predicts that H_2_ from renewables in combination with electrolysis will cost between 1.6 and 4.0 $/kg around the globe^7^. To reach these scenarios, it is crucial to increase the solar-to-H_2_ (STH) conversion efficiency well beyond 15%^8^ in addition to reducing the system CAPEX and increasing operating hours^7,8^. Systems using sunlight concentration in combination with highest performance photoabsorbers and electrocatalysts facilitate these targets^9^.

Different approaches have been reported in the literature to convert sunlight into hydrogen. Artificial photosynthesis, such as photobiological water splitting, in which algae produce H_2_ rather than oxygen (O_2_) gas leads to low conversion efficiencies in the range of 1% and consequently require large areas for producing relevant amounts of H_2_^10^. Photocatalytic processes are more efficient if the light absorber provides a potential difference exceeding 1.23 V and absorbs a wide range of photon energies of the solar spectrum. For example, a GaInP/GaAs tandem solar cell coated with TiO_2_ and Ni based catalysts reached a STH conversion efficiency of 10.5% when submerged in a 1.0 M KOH aqueous solution^11^. Also, GaInP/GaInAs tandem-structures with functionalized layers and embedded Rh and RuO_2_ catalysts have been used to split water with an STH conversion efficiency between 14 and 17.4%^12^. Furthermore, three years later the same research group raised the STH conversion efficiency of such device to 19.3% by reducing the optical and electrical losses^13^. These devices provide sufficiently high voltages to electrolyze water and have also been used under concentrated light.

An alternative approach was developed by our research group using tandem solar cells in a hybrid photovoltaic/electrolysis module where the solar cells are directly connected to the cathode and anode of the electrolysis cell^14–17^. A system using eight dual-junction GaInP/GaInAs concentrator photovoltaic (CPV) cells at 252 suns concentration, independently connected to eight polymer electrolyte membrane (PEM) electrolysis cells, reached an average STH conversion efficiency of up to 19.8% at current densities beyond 800 mA/cm^2^. This result was obtained from outdoor measurements on a dual-axis solar tracker^17^. Using triple-junction (3 J) solar cells increases the voltage but reduces the current and in consequence also the H_2_ generation. Hence, a different interconnection scheme between solar and electrolysis cells must be used to reach high STH conversion efficiencies^18–20^. One report used a GaInP/GaAs/GaInNAsSb 3 J solar cell at 42 suns to drive two series-interconnected PEM electrolysis cells at current densities of up to 29 mA/cm^2^. This resulted in an STH conversion efficiency of 30% measured indoors under a solar simulator (AM1.5 direct spectrum) with controlled temperatures (T_solar cell_ = 25 °C and T_electrolysis cells_ = 80 °C)^19^. At higher input powers ( > 2 kW) a parabolic dish concentrated the direct normal irradiance (DNI) ca. 800x onto a solar reactor consisting of a 3 J III-V PV module and an embedded PEM stack to split water at a STH conversion efficiency of 24.4% while cogenerating heat at 35.5% efficiency^21^.

In this work, we explore the potential of using four-junction (4 J) solar cells under 226× geometrical concentration to drive two series connected PEM electrolysis cells. A prototype of this technology was built and measured outdoors in Freiburg, Germany under outdoor prevailing conditions. This hybrid module called HyCon® reached a record STH conversion efficiency of 31.3%.

## Results

### The HyCon® technology: a CPV/PEM electrolysis module

**Fig. 1:**
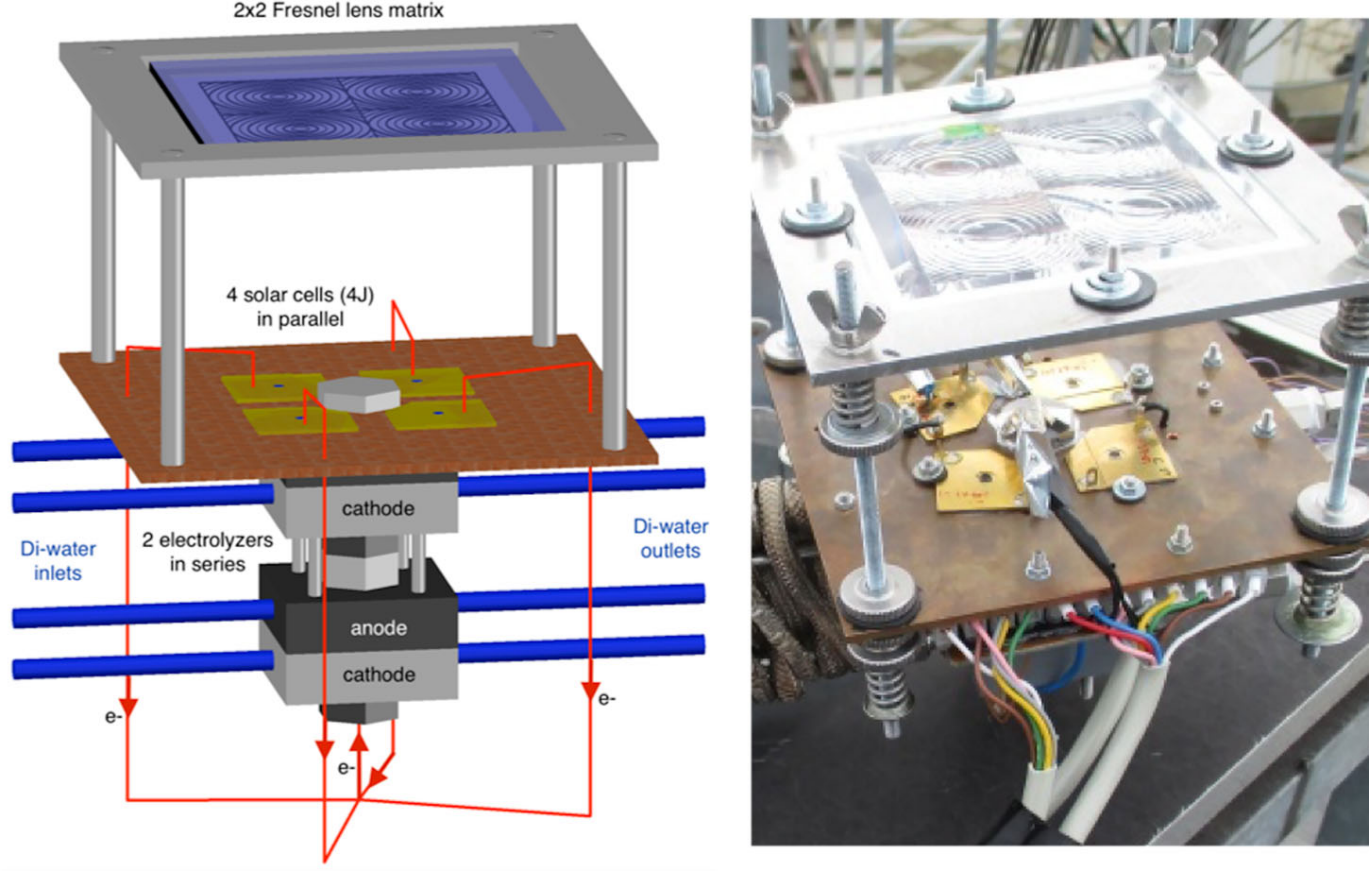
Visualization images of the HyCon® module developed in this work. The figure shows a schematic (left) and a photograph (right) of the HyCon® concentrator photovoltaic/electrolysis module equipped with 4 four-junction (4 J) solar cells in the focus of four Fresnel lenses. The back side of the solar cells array is electrically connected to the top anode of two series interconnected PEM electrolysis cells and their front side to the bottom cathode. The prototype system has a total aperture area for the collection of sunlight of 64 cm^2^.

A schematic and a photograph of the CPV/PEM electrolysis HyCon® module developed in this work is shown in Fig. 1 (left) and (right), respectively. It consists of a 2 × 2 array of Fresnel lenses (16 cm^2^ each) and four parallel-interconnected 4 J CPV cells (7 mm^2^ each), which are electrically and thermally connected to the anode and cathode of two series-interconnected PEM electrolysis cells. These solar and electrolysis cells are mounted on the front and back side of a copper plate which serves as the mechanical support for the structure.

As observed in Fig. 1, the array of Fresnel lenses is held by an aluminum frame at a focal distance of 80 mm relative to the 4 J CPV cells. Screws allowed a fine adjustment of the lens to cell distance. Solar cells were mounted on four Cu substrates (29 × 29 mm^2^) which are placed onto a large Cu baseplate (15 × 15 cm^2^). The stack of two PEM electrolysis cells interconnected in series was mounted on the rear side of the Cu baseplate where both anodes and cathodes have an inlet and an outlet to feed the membrane with deionized water and to flush out the reaction products (H_2_ and O_2_). Here, four Ti screws were used to conduct the current to the cathode and from the anode of both electrolysis cells. The top anode screw which is in contact with the Cu baseplate serves as the electrical and thermal interconnection between the 4 J CPV cell array and the PEM electrolysis stack. Further details about the outdoor characterization of the HyCon® module are given in the methods section.

### The four-junction (4 J) concentrator photovoltaic (CPV) cell

**Fig. 2:**
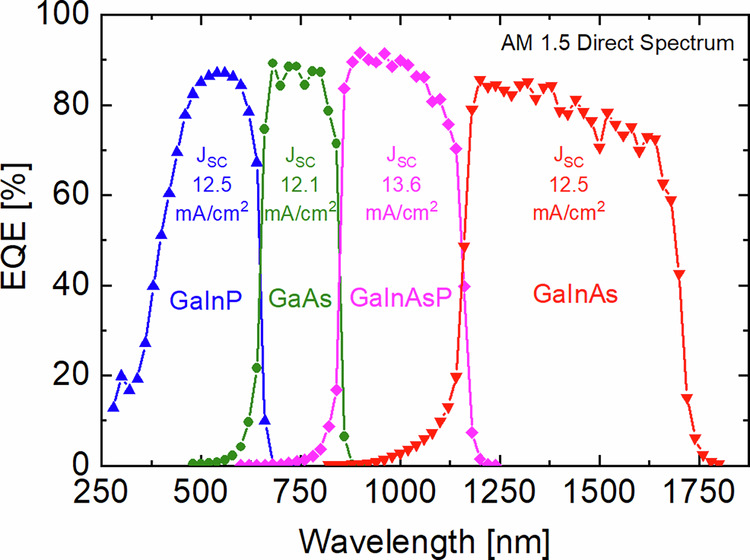
Measured external quantum efficiency (EQE) of a GaInP/GaAs//GaInAsP/GaInAs wafer-bonded four-junction solar cell. The plot shows the photogenerated current density (J_SC_) in each sub-cell as a function of wavelength under the reference AM1.5 direct spectrum.

The 4 J solar cells used in this work are realized by wafer-bonding (//) of two dual-junction structures, i.e. GaInP/GaAs//GaInAsP/GaInAs^22,23^. The GaInP/GaAs tandem device is epitaxially grown on a GaAs substrate as a lattice-matched inverted structure, whereas the GaInAsP/GaInAs is grown as an upright lattice-matched structure on InP. This yields a 4 J solar cell with energy band gaps of 1.89, 1.42, 1.12 and 0.74 eV, which are very close to the theoretical optima under the concentrated reference AM1.5 direct spectrum, i.e., 1.94, 1.44, 1.04 and 0.69 eV^24^. Evidence of that is given in Fig. 2, where the external quantum efficiency (EQE) of the device is shown together with photogenerated current densities. The fact that they are close together is important because the sub-cell with the lowest current density limits the overall device and consequently the amount of hydrogen that can be generated. This 4 J solar cell technology has demonstrated world record solar-to-electricity (STE) conversion efficiencies of up to 47.6% under the concentrated reference AM1.5 direct spectrum^25,26^. Further details about the 4 J solar cell fabrication are given in the methods section.

### The polymer electrolyte membrane (PEM) electrolysis cell

**Fig. 3:**
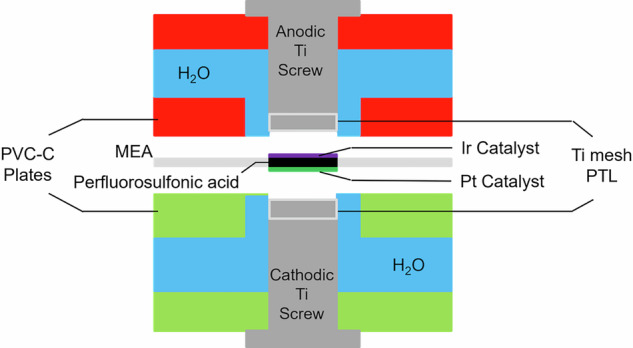
Schematic blown-up cross section view of the PEM electrolysis cell used in this work. The anode and cathode plates made of chlorinated polyvinyl chloride (PVC-C) enclose the perfluorosulfonic acid (PFSA) membrane electrode assembly (MEA) where deionized water is fed to the Ir and Pt catalyst layers through the Ti meshes which act as porous transport layers (PTL).

One out of the two PEM electrolysis cells used in this work is schematically shown in Fig. 3 and it corresponds to the same cell described in ref. ^27^ as Gen II. Its enclosure consists of two machined chlorinated polyvinyl chloride (PVC-C) plates which bring the anodic and cathodic deionized water (di-water) flows to the reaction chamber where the membrane electrode assembly (MEA) is located. The latter is a commercial perfluorosulfonic acid (PFSA) membrane with a thickness of 175 μm and a circular active area of 1.13 cm^2^ which is coated with iridium and platinum as catalysts on the anode and on the cathode side, respectively. Through the middle section of each PVC-C plate there is a hole to insert a Ti screw which slightly presses down a Ti mesh onto the MEA to serve as a porous transport layer and as a flow field for the deionized water. Further details about the electrolysis cells fabrication and preconditioning are given in the methods section.

### Configuration of CPV cell array and PEM electrolysis stack

**Fig. 4:**
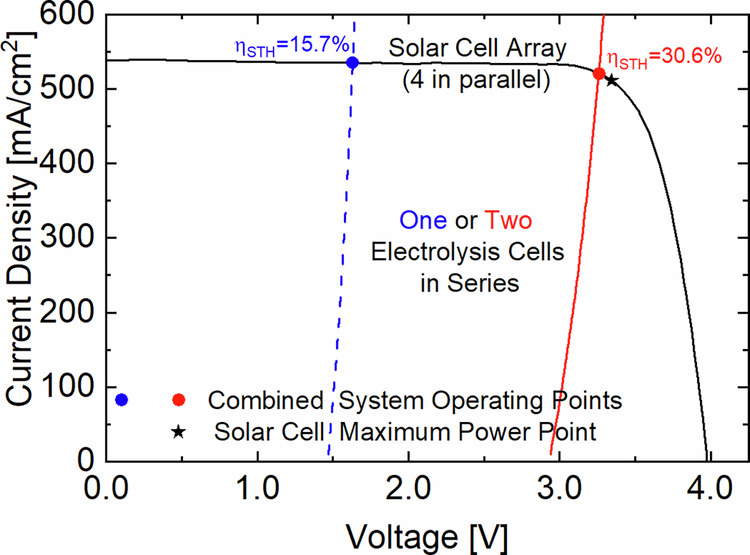
Measured J-V curves of the 4 J concentrator PV (CPV) cell array and of the PEM electrolysis cells. The plot shows the J-V curves of the 4 J CPV cell array (black solid line), a single PEM electrolysis cell (blue dashed line) and a stack of 2 PEM cells interconnected in series (red solid line). The maximum power point (black star) corresponds to the current and voltage pair where the solar cell delivers the maximum power. The intersection points (blue and red dots) correspond to the operating points of the two possible system combinations, and they determine the solar-to-H_2_ (STH) conversion efficiency. As the sizes of the solar and electrolysis cells are different, both current densities were normalized with the active area of the membrane electrode assembly which is where H_2_ is generated, i.e., 1.13 cm^2^.

Direct coupling of a solar cell with PEM electrolysis means that the latter will define the operating point along the current density-voltage (J-V) curve of the former. Ideally, the desired working point of the electrolysis cell should match the maximum power point (MPP) of the solar cell to achieve the highest STH conversion efficiency of the combined system. Figure 4 shows the J-V curve of the four 4 J solar cells in parallel measured outdoors (DNI = 886 W/m^2^ and concentration = 226x) and the indoor measured polarization curves of one and two PEM electrolysis cells in series at 60 °C. As denoted by the intersection of the single PEM cell (blue dashed line) with the 4 J CPV cell array (black solid line), the operating point would be 1.71 V below V_MPP_. Hence, the STH conversion efficiency would remain at 15.7%. A combination of two PEM cells in series (red solid line) with the 4 J CPV cell array is the optimum fit, as the operating point (red dot) would barely remain 79 mV below V_MPP_. In this case, the achievable STH conversion efficiency would increase to 30.6%.

The solar-to-H_2_ conversion efficiency (η_STH_) corresponds to the product of the CPV array efficiency at the operating point multiplied with the efficiency of the PEM electrolysis stack. The resulting expression is given in Eq.( 1),

1 $${\eta }_{{STH}}=\frac{{I}_{{OP}}\cdot {V}_{{th}}\cdot {N}_{S}}{{A}_{{lens}}\cdot {DNI}}\cdot {\eta }_{F}$$

where I_OP_ is the current at the operating point, V_th_ = 1.48 V is the thermo-neutral voltage at standard conditions, N_S_ = 2 is the number of electrolysis cells interconnected in series, A_lens_ = 64 cm^2^ is the aperture area of the lens array through which the DNI is concentrated and η_F_ is the Faraday efficiency. The latter accounts for how effectively the electrons contribute to splitting water given that in practical electrolysis cells stray currents can occur as the membrane has a finite resistance. Another source of Faradaic losses is the recombination of H_2_ and O_2_ at the opposite electrode as both gases have a small but not zero probability to permeate through the membrane. We characterized the Faraday efficiency of the electrolysis stack in the lab and found that at a current density of 250 mA/cm^2^, the losses were as low as 1.3%. This is a typical value for such a PFSA membrane operating under atmospheric conditions. Since the Faradaic losses remain nearly constant, increasing the operating current density decreases their relative contribution. Therefore, we chose to interconnect four 4 J CPV cells in parallel to boost the current density of the electrolysis cells beyond 500 mA/cm^2^ during outdoor operation. Doubling the operating current density increases the Faraday efficiency beyond 99.4%. Further details about the Faraday efficiency characterization are given in the methods section.

### Dynamic behavior of the operating point

**Fig. 5:**
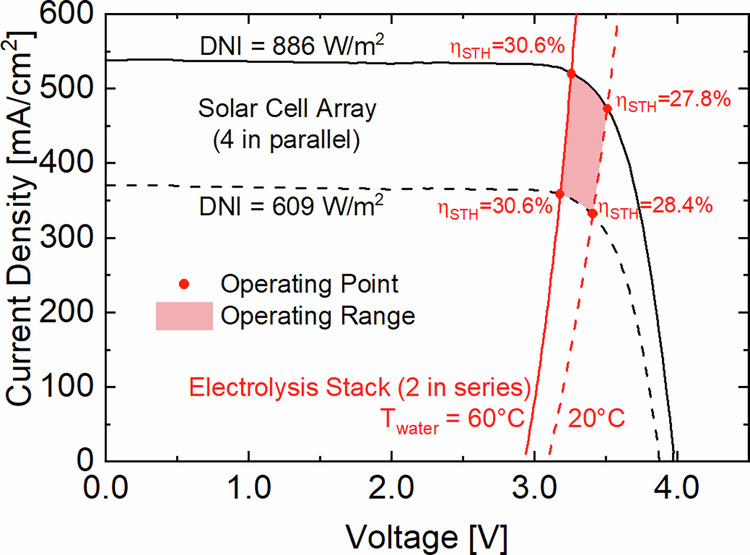
Measured J-V curves of the 4 J concentrator PV (CPV) cell array at different irradiance levels and of the PEM electrolysis stack at different temperatures. The plot shows the J-V curves of the 4 J CPV cell array under 886 W/m^2^ (black solid line) and 609 W/m^2^ (black dashed line) intensities of direct normal irradiance (DNI) and of the PEM electrolysis stack at 60 °C (red solid line) and 20 °C (red dashed line) water temperature. The intersections between the CPV cell array and the PEM electrolysis stack curves (red dots) which occur within the operating range (red shaded area) determine the operating points and the respective solar-to-H_2_ (STH) conversion efficiencies of the system. As the sizes of the solar and electrolysis cells are different, both current densities were normalized with the active area of the membrane electrode assembly which is where H_2_ is generated, i.e. 1.13 cm^2^.

So far, the operation of the HyCon® module was explained with a single intersection of the superimposed J-V curves of the solar and electrolysis cells which were measured separately at a fixed DNI intensity (886 W/m^2^) and water temperature (60 °C). However, the operating point is highly dynamic as the J-V curves are particularly influenced by DNI and temperature changes. As depicted in Fig. 5, when the DNI decreases from 886 W/m^2^ (black solid line) to 609 W/m^2^ (black dashed line) the current density of the solar cells decreases linearly and their voltage logarithmically. Hence, the H_2_ generation drops by 30.4%_rel_ when the DNI decreases by 31.2%_rel_. In contrast, when the water temperature is reduced from 60 °C (red solid line) to 20 °C (red dashed line) the voltage required for the electrolysis increases.

As depicted by the operating points (red dots), a 40 K drop in water temperature reduces the STH conversion efficiency up to 2.8%_abs_. This is because the electrolysis overvoltage increases and forces the operating point down the J-V curve of the solar cells. For this reason, it is desired to operate the electrolysis stack at a higher temperature to maintain the operating voltage at or below the V_MPP_ of the solar cell array regardless of the illumination intensity. Hence, our design aimed at thermally coupling the CPV array with the PEM electrolysis stack to increase the operating temperature of the MEA. However, the prototype in this work only raised it by 2 K, thus, we used active water heating at the inlet.

**Fig. 6:**
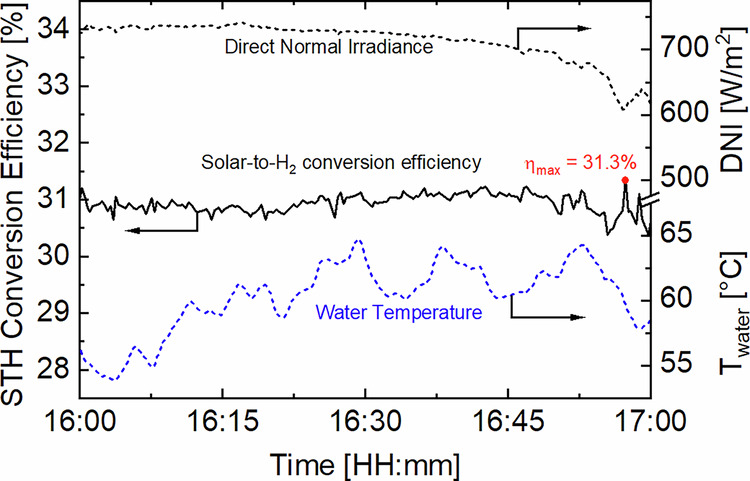
Measured key performance metrics of the HyCon® module as a function of time. The plot shows the time-series of direct normal irradiance (DNI), solar-to-H_2_ (STH) conversion efficiency, and electrolysis stack water temperature of the HyCon® module for a typical summer hour in Freiburg, Germany. The red dot denotes the maximum STH conversion efficiency achieved by the HyCon® module, i.e., 31.3%, according to the higher heating value of H_2_ and to a Faraday efficiency of η_F_ = 99.4%. No performance degradation over time was observed throughout the 13 days of testing ( > 13,000 measurements) where the STH conversion efficiency fluctuated between 25 and 31.3% due to changes in the water temperature and in the atmospheric conditions.

Outdoor testing of the CPV/PEM electrolysis module on a dual-axis solar tracker over 13 summer days in Freiburg, Germany demonstrated that it is possible to generate hydrogen with a STH conversion efficiency of up to 31.3%. This record value was achieved with efficiencies of 34.7 and 91.1% for the CPV array and for the PEM electrolysis stack at the operating point, i.e., 368 mA/cm^2^ and 3.25 V. Figure 6 shows a representative hour during which the STH conversion efficiency remained continuously above 31% while the DNI changed from 738 to 608 W/m^2^. During this time period, the average temperature of the water ( ± its standard deviation) fed to the PEM electrolysis stack was (61 ± 3)°C. As previously shown in Fig. 5, keeping the operating point below the MPP voltage (V_MPP_) by pre-heating the water favors the STH conversion efficiency.

Regarding daily performance during a typical diurnal cycle without assisted heating, the water temperature increased from 20 to 31 °C, whereas the STH conversion efficiency quickly raised from 25% at medium DNI (540 W/m^2^) in the morning to 30% at high DNI (880 W/m^2^) before decreasing again with the drop of DNI in the evening. In these 11 hours of operation our module generated 61.6 g of H_2_/m^2^ of lens aperture area, which correspond to a daily STH conversion efficiency of 28.8%. Regardless of the low water temperature, the PEM cells always operated at low voltages below 1.8 V, thus maximizing photocurrent utilization and avoiding lifetime shortening by accelerated degradation. This operating voltage is typical for the membrane’s beginning of life at 2 A/cm^2^. With assisted heating the water temperature increased from 20 to 57 °C and yielded a daily STH conversion efficiency of 29.5%. Comparing these results to an equivalent PEM stack running 24/7 at the same daily average efficiency of 87% (i.e. 1.7 V/cell) powered by a concentrator PV module equipped with the same type of 4 J solar cells that has a rated efficiency of 36.7%^28^. yields a capacity factor of 31.6% in Freiburg. This is 5% higher than the best photovoltaic/electrolysis systems reported in literature which range between 20 and 30%^29–31^.

Concerning reliability of PEM water electrolysis cells, it is well known that operating them at high voltages ( > 2.4 V) increases the catalyst layer degradation due to the higher electrochemical stress induced mainly at the anode by the larger overpotential. For instance, high performance PEM cells usually degrade at a rate below 10 µV/h, whereas typical ones lose between 10 and 20 µV/h. This gives them useful lifetimes of 40,000 to 60,000 operating hours^32^. Although we used an ion exchanger in our outdoor setup, the low conductivity of the water cannot be guaranteed as in an indoor test bench, hence the degradation rate could be expected to go beyond the above-mentioned values. Nevertheless, no degradation was observed during the 107 hours of operation in which our system went through 13 dynamic cycles. Throughout this time, the voltage and current density fluctuated between 0.2 and 0.5 A/cm^2^ and 1.6–1.8 V/cell, whereas the water temperature fluctuated between 17 and 70 °C.

As far as reactant supply goes, we varied the water flow between 5 and 15 ml/min using an electric pump, but we also allowed the system to operate at lower volume flows under natural convection during some of the days, observing a higher temperature increase of the water at lower volume flow.

## Discussion

The STH conversion efficiency of our HyCon® module, calculated with Eq. 1, disregards the water preheating to 60 °C because there is enough heat remaining in the solar cells to go even higher in a closed-loop system. Hence, active heating will be avoided through an enhanced thermal coupling between the CPV and electrolysis cells in a future design. An example calculation of such a system is given in the methods section. Given the current system configuration, feeding water at higher temperature (80 °C) would further increase the electrolysis stack efficiency. However, H_2_ generation would only increase marginally as there is little current density to be gained by further reducing the operating voltage. Nevertheless, this would enable the use of higher irradiance concentrations on the CPV cells to boost the current density, while enabling semiconductor material savings at nearly the same STH conversion efficiency. In consequence, more solar H_2_ could be generated for the same Ir and Pt loading of the electrodes which should further reduce the cost per kg of H_2_. In fact, this is the approach followed by the company Fusion-Fuel which uses a large Fresnel lens to concentrate the DNI 1400 times onto a 3 J CPV cell that is directly coupled to a PEM electrolysis stack^33^.

**Fig. 7:**
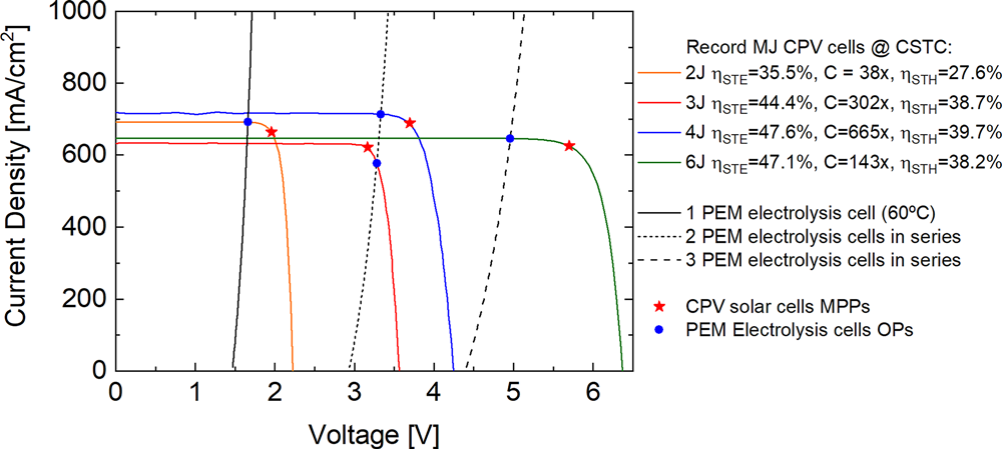
Scaled J-V curves of the world record III-V multi-junction (MJ) concentrator PV (CPV) cells and of a series arrays of PEM electrolysis cells. The plot shows the J-V curves at concentrator standard test conditions (CSTC)^40^ of the parallel arrays of eleven 2 J GaInAsP/GaInAs (orange)^34^, one 3 J GaInP/GaAs/InGaAs (red)^35^, two 4 J GaInP/GaInAs//GaInAsP/GaInAs (blue)^26^ and six 6 J AlGaInP/AlGaAs/GaAs/GaInAs(3) (green)^36^ world record MJ CPV cells and of 1 (black solid), 2 (black dotted) and 3 electrolysis cells in series (black dashed) at 60 °C. All current densities were normalized with the active area of the membrane electrode assembly, i.e., 1.13 cm^2^. The red stars correspond to the maximum power points (MPPs) of the CPV cell arrays, whereas the blue circles denote the operating points (OPs) of the directly coupled PEM electrolysis stack. The solar-to-electricity (STE) conversion efficiency and concentration (C) level of each solar cell array, as taken from its reference, and the solar-to-H_2_ (STH) conversion efficiency of each CPV/PEM electrolysis cell combination, assuming η_opt_ = η_F_ = 100%, are given in the legend.

To assess the potential of directly coupled CPV/PEM electrolysis systems, Fig. 7 shows the J-V curves of world record multi-junction (MJ) CPV cells with 2, 3, 4 and 6 junctions. It was calculated how these cells could be coupled to 1, 2 or 3 PEM electrolysis cells in series to achieve maximum STH conversion efficiency. For each case, a parallel array of solar cells delivering 700-800 mA to the 1.13 cm^2^ MEA at the concentration at which the world record was achieved is shown. The comparison neglects optical and faradaic losses and uses the scaled J-V curve of the electrolysis stack from this work operating at 60 °C and between 1.64 to 1.67 V/per cell. The latter was chosen because within this narrow range the electrolysis efficiency remains nearly constant at (89.6 ± 0.7)% and the Faraday efficiency approaches 100% at these operating current densities (600-700 mA/cm^2^). Thus, the STH conversion efficiency depends only on the maximum current that can be generated by the CPV cell when connected directly to the electrolysis cells. As shown in Fig. 7, a parallel array of eleven GaInAsP/GaInAs 2 J CPV cells under 38x concentration^34^ would be able to run a single PEM electrolysis cell below its V_MPP_ to achieve a η_STH_ = 27.6%. In contrast, a single GaInP/GaAs/InGaAs 3 J CPV cell under 302x concentration^35^ would be able to run two PEM electrolysis cells in series at η_STH_ = 38.7%. In this case, the STH conversion efficiency is considerably increased due to the higher efficiency of the 3 J solar cell and despite its operation beyond V_MPP_. Also running two PEM electrolysis cells in series, a parallel array of two of the next generation GaInP/GaInAs//GaInAsP/GaInAs 4 J CPV cells used in this work under 665x concentration^23,26^ reaches the highest potential to convert sunlight into H_2_ at an efficiency of 39.7%. The improvement over the result of this work is explained by a 5.6% higher current resulting mainly from a higher photon-absorption due to lower metallization shading and an enhanced four-layer antireflective coating. Translating this improvement to outdoor operating conditions which are subjected to additional optical ( ~ 15%_rel_) and thermal ( ~ 2%_abs_) losses would yield a STH conversion efficiency around 33%. Lastly, a parallel array of six AlGaInP/AlGaAs/GaAs/GaInAs(3) 6 J CPV cells under 143x concentration^36,37^ would be able to run three PEM electrolysis cells in series at η_STH_ = 38.2%. Hence, today’s highest STH conversion efficiency is achieved by 4 J CPV cells coupled to two series connected PEM electrolysis cells. This is because the dynamic operating voltage of the latter maximizes the current density utilization of the former. In other words, operating as close as possible to the MPP of the solar cells is always desired because below J_MPP_ photocurrent is wasted, i.e. lower H_2_ generation, whereas below V_MPP_ voltage which could be transformed into photocurrent by adjusting the band gaps of the MJ cells is not used.

Summarizing, this article presents the outdoor performance of a CPV/PEM electrolysis module that directly interconnects a parallel array of four 4 J CPV cells with two PEM electrolysis cells in series. The device converts sunlight into H_2_ with up to 31.3% efficiency according to the HHV of the latter. The STE conversion efficiency of the four parallel connected CPV cells at the operating point was 34.7%, whereas the PEM electrolysis stack operated at 91.1% efficiency with reference to the thermo-neutral cell voltage at standard temperature and pressure. To our knowledge, this is the highest reported efficiency of a solar H_2_ generating system measured under outdoor operating conditions, where electrical losses due to temperature ( ~ 2%_abs_) and optical losses due to the concentrator lenses ( ~ 15%_rel_) are present and considered. Fluctuations in the solar spectrum throughout the day and seasons have an influence on the current matching between the sub-cells of the MJ solar cells and therefore on the current which drives the electrolysis reaction. During our experiments we quantified this effect had an impact of approximately ±3%_rel_ on the STH conversion efficiency. Hence, our results show that using multi-junction solar cells under concentration directly interconnected with PEM electrolysis cells can reach the highest conversion of sunlight into H_2_.

From a broader perspective, CPV intrinsically enables module efficiency beyond 35% by using multi-junction solar cells, and increases the temperature of unused heat above 70 °C due to light concentration surpassing 200x. This is considerably above what any flat-plate PV technology can reach today and highly beneficial to produce sustainable hydrogen via the direct coupled HyCon® concept. Especially in high DNI regions of Mexico, USA, Chile, Australia, Africa, and the Middle East, CPV could reach levelized costs of electricity (LCOE) in the range of 15-25 $/MWh which would enable competitive decentralized hydrogen generation for energy storage in remote locations. In addition, the HyCon® system does not require electronics for power conversion, thus, particularly reducing the balance of system costs and enabling complete independent operation from the grid.

According to the levelized cost of hydrogen (LCOH) analysis performed by the Clean Air Task Force (CATF)^30^ and the CPV LCOE estimated by us (15-25 $/MWh) in combination with the HyCon® capacity factor reached in this work (31.6%), the HyCon® could yield unsubsidized green H_2_ costs in the range of 3 $/kg. This value aligns with the lower boundary estimated by the CATF and falls well within the range projected by the IEA (1.6–4 $/kg). Further reduction to 2 $/kg would require capacity factors above 60%, clean electricity for less than 10 $/MWh or a less stringent combination of both.

In addition, although CPV modules do not convert diffuse irradiance into electricity, they are capable to reach 20–40% higher energy yields^38^ than state-of-the-art bifacial single-axis tracked flat-plate PV modules in high DNI regions where green H_2_ is expected to be produced and exported from. Furthermore, equipping the former with a diffuse light absorber as a heat dissipating substrate could increase their energy yield by up to 10% in those same places^38^. Hence, potentially increasing the capacity factor of HyCon® technology to 35% to reach LCOH below 3 $/kg.

## Methods

### Four-junction (4 J) solar cell fabrication

The four-junction concentrator solar cells used in this work were produced by a combination of epitaxial growth (using metal-organic-vapor-phase epitaxy in an Aixtron 2800-G4 reactor with 8 × 4” substrate configuration) and wafer bonding. The GaInP/GaAs top- and the GaInAsP/GaInAs bottom-tandem cells were grown on GaAs and InP, respectively. Typical growth conditions were applied, such as temperatures between 600 and 650 °C, a reactor pressure of 50 mbar and V/III ratios of 20–60. The top cell structure was joint with the bottom cell structure using wafer bonding before removing the GaAs substrate and processing top and bottom contacts, as well as anti-reflective coatings. More details on the fabrication of these 4 J CPV cells can be found in references^22,23^.

### PEM electrolysis cell fabrication and Faraday efficiency characterization

Commercially available catalyst coated membranes based on Nafion® 117 PFSA from Chemours^TM^ were used in this work. Their anode was coated with Ir and their cathode with carbon supported Pt. For the fabrication of the electrolysis cells, the MEAs were enclosed between two PVC-C plates. Screwing M14 Ti screws through the PVC-C plates, the Ti meshes which serve as a porous transport layer were carefully pressed against the active area of the MEAs. These meshes were cut out from a 0.5 mm thick and 73% porous sheet of Ti fiber sinter by water jet. The assembled electrolysis cells were preconditioned in a thermal and galvanostatic manner by passing water through them at 15 ml/min and 60 °C, while applying a current density of 0.2 A/cm^2^ for 30 min followed by 1 A/cm^2^ for another 30 min After activation of the ionic conductivity of the MEAs by preconditioning, it was observed that electrolysis started at 55 mV above the thermo-neutral cell voltage and that no major concentration losses due to catalyst layer saturation were present up to 3 A/cm^2^. The Faraday efficiency of the electrolysis cells was characterized in the lab through a gas purity measurement. The measurement yielded the presence of 140 ppm of O_2_ on the H_2_ side for a fluidic interconnection of four cells in parallel that were operated electrically in series at 250 mA/cm^2^. At 100% hydrogen humidity (which represents the worst-case scenario for collected H_2_ volume), the Faraday efficiency was measured to be 97.4% for the electrolysis array. Therefore, the Faraday efficiency of a single electrolysis cell corresponds to 99.4% at 250 mA/cm^2^.

### CPV/PEM electrolysis module characterization under real operating conditions

Evaluation of the CPV/PEM electrolysis module performance under real operating conditions was carried out on a high-precision ( < 0.2°) dual-axis solar tracker during the summer in Freiburg, Germany. The tracker was equipped with two pyrheliometers that measure the direct normal irradiance with an uncertainty below ±2% and with a portable weather station to measure the ambient temperature and the wind velocity. In addition, a 2 L tank with four outlets containing di-water served as the gas-water separator and it was mounted at the top of the tracker. Two outlets are for natural convection flow operation and go directly to an ion exchanger before reaching the anode and cathode of the electrolysis stack and the other two are for forced convection flow operation and they pass through a water pump before following the same path. Hence, the electrolysis cells were always fed with high quality di-water ( ~ 0.1 µS/cm) at volume flows between 5 and 45 ml/min and at atmospheric pressure (ca. 1 bar). The tank also has two inlets, one for the return of the di-water mixed with H_2_ and the other for the di-water and O_2_ mixture. Also, the tank has two holes at the top which are not tightly sealed to allow venting of the produced gases. Furthermore, a resistive heater mounted on the inlet pipes of the electrolysis cells allowed to heat the water up to 21 K above ambient temperature during testing. This was measured with Pt100 resistor thermometers. The operating voltage and current of the electrolysis cells were measured using voltage sensors and a shunt resistor. The reported water temperature corresponds to the arithmetic mean of the top anode inlet and all four outlet measurements. The operating voltage consists of the sum of the voltages of both electrolysis cells because they were measured independently. The operating current was calculated from the voltage drop measured across the 20 mΩ shunt resistor which was connected in series with the electrolysis stack. All these parameters were recorded continuously every 15 seconds using an analog data logger.

### Heat availability on the solar cells and potential transfer to the water flow

At concentrator standard operating conditions (DNI = 900 W/m^2^ and T_amb_ = 20 °C) the 4 J solar cells inside a closed CPV module using 16 cm^2^ lenses typically operate above 70 °C and convert around 34% of the DNI into electricity. If an additional 15% is subtracted from the remaining 66% due to the optical losses, 459 W/m^2^ are still available as heat on the solar cells. Collecting this heat could increase the temperature of a 148 ml/min flow of water from 20 to 60 °C at a heat exchange efficiency of 90%. That is enough water to sustain electrochemical water splitting at 1 A/cm^2^ in 37 PEM stacks each equipped with four 2 cm^2^ electrolysis cells electrically and fluidically in series. For a 1 m^2^ HyCon® module only 22 PEM stacks are necessary when each one of them is directly coupled to an array of two strings in series of fourteen 4 J solar cells in parallel. Moreover, to heat up effectively the water flow to 60 °C, the gradient to the solar cells should be around 10 K which requires a thermal resistance between them smaller than 0.022 K/(W/m^2^). Even with a 4 mm glass layer in between, the thermal resistance would remain well below at 0.004 K/(W/m^2^). Hence, passing the water flow through a small metal channel directly attached to the glass substrate underneath the solar cells of a CPV module like the FLATCON® ^39^ would bring the water to the desired operating temperature. In addition, this would reduce the temperature of the solar cells and it would increase their voltage to enable a higher utilization of photocurrent to produce H_2_. Furthermore, regulating the water flow would allow to keep the operating temperature stable as the DNI and ambient temperature vary throughout the day. Rounding up, the PEM stack and the CPV module could be fabricated independently from each other to be only coupled electrically and fluidically in situ upon deployment. Also, different system configurations to enable operation at higher current densities or the use of larger electrolysis cells would be possible, as this is just an example calculation to show that the required electricity and heat to split water efficiently are available in the HyCon® system.

### Ethics declarations

HyCon® is a registered trademark protected under U.S. Patent No. 2007246370 AA, titled “Device and method for photovoltaic generation of hydrogen”, with FD listed as inventor. All other authors declare no competing interest.

## Data Availability

The data sets generated and analyzed in this study are available from the corresponding author on reasonable request.
